# Training Language Models for Estimating Priority Levels in Ultrasound Examination Waitlists: Algorithm Development and Validation

**DOI:** 10.2196/68020

**Published:** 2025-07-22

**Authors:** Kanato Masayoshi, Masahiro Hashimoto, Naoki Toda, Hirozumi Mori, Goh Kobayashi, Hasnine Haque, Mizuki So, Masahiro Jinzaki

**Affiliations:** 1 Department of Radiology, School of Medicine, Keio University, 35 Shinanomachi, Shinjuku-ku, Tokyo 160-8582, Tokyo, Japan, 81 3-3353-1211 ext 62477; 2 GE Healthcare Japan, 4-7-127, Asahigaoka, Hino, Tokyo, Japan

**Keywords:** natural language processing, clinical informatics, large language model, machine learning, health resources, ultrasonography, hospital information systems.

## Abstract

**Background:**

Ultrasound examinations, while valuable, are time-consuming and often limited in availability. Consequently, many hospitals implement reservation systems; however, these systems typically lack prioritization for examination purposes. Hence, our hospital uses a waitlist system that prioritizes examination requests based on their clinical value when slots become available due to cancellations. This system, however, requires a manual review of examination purposes, which are recorded in free-form text. We hypothesized that artificial intelligence language models could preliminarily estimate the priority of requests before manual reviews.

**Objective:**

This study aimed to investigate potential challenges associated with using language models for estimating the priority of medical examination requests and to evaluate the performance of language models in processing Japanese medical texts.

**Methods:**

We retrospectively collected ultrasound examination requests from the waitlist system at Keio University Hospital, spanning January 2020 to March 2023. Each request comprised an examination purpose documented by the requesting physician and a 6-tier priority level assigned by a radiologist during the clinical workflow. We fine-tuned JMedRoBERTa, Luke, OpenCalm, and LLaMA2 under two conditions: (1) tuning only the final layer and (2) tuning all layers using either standard backpropagation or low-rank adaptation.

**Results:**

We had 2335 and 204 requests in the training and test datasets post cleaning. When only the final layers were tuned, JMedRoBERTa outperformed the other models (Kendall coefficient=0.225). With full fine-tuning, JMedRoBERTa continued to perform best (Kendall coefficient=0.254), though with reduced margins compared with the other models. The radiologist’s retrospective re-evaluation yielded a Kendall coefficient of 0.221.

**Conclusions:**

Language models can estimate the priority of examination requests with accuracy comparable with that of human radiologists. The fine-tuning results indicate that general-purpose language models can be adapted to domain-specific texts (ie, Japanese medical texts) with sufficient fine-tuning. Further research is required to address priority rank ambiguity, expand the dataset across multiple institutions, and explore more recent language models with potentially higher performance or better suitability for this task.

## Introduction

### Waitlist System

Ultrasound, a noninvasive imaging modality, enables real-time visualization of organs and blood flow and can be performed safely in pediatric and obstetric populations. However, imaging quality depends on the proficiency of the technician. Most hospitals implement reservation systems that allocate available slots to physicians due to a shortage of ultrasound technologists. Frequently, slots for the immediate future are fully booked, and these systems typically lack mechanisms for automatic urgency assessment.

Our hospital has implemented a waitlist system in case an appointment is canceled, and a slot becomes vacant. The system prioritizes examination requests based on urgency and clinical value. This approach facilitates more efficient use of canceled slots, reducing patient wait times, minimizing hospital stays, and improving overall care quality.

**Figure 1.**
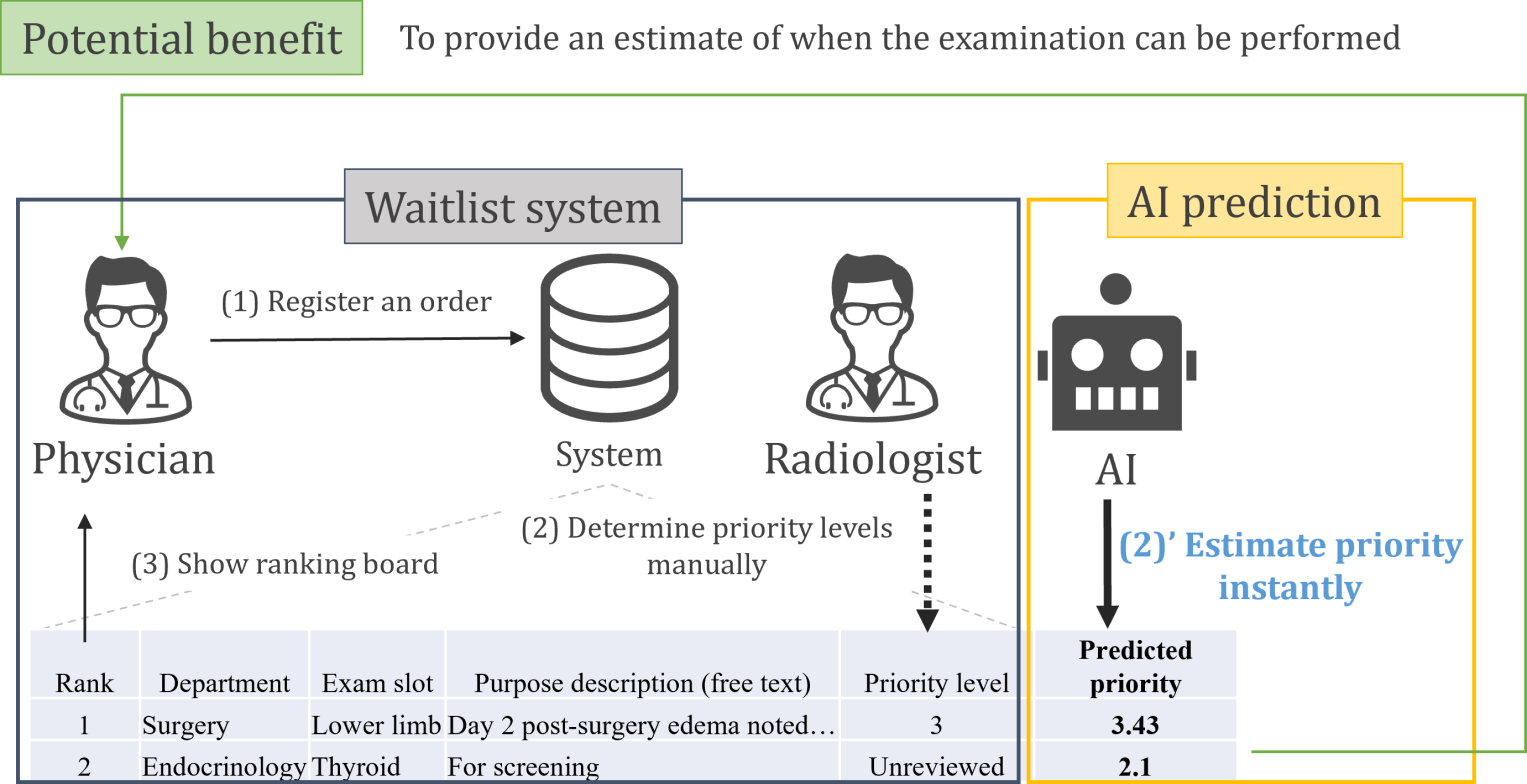
Artificial intelligence-predicted priority levels will allow physicians to estimate waiting time before the official priority is determined by radiologists. AI: artificial intelligence.

Our waitlist system organizes examination requests into 6 priority tiers, determined by board-certified radiologists based on the examination purpose, which is recorded as a brief free-text entry by the requesting physician (Figure 1). The waitlist is accessible to all physicians, enabling them to anticipate when their orders might be processed. However, the delay in updating until radiologists complete their reviews has led to difficulties in providing real-time wait time estimates. Therefore, we investigated the potential of artificial intelligence (AI) language models to provide preliminary priority estimations.

### Use of Language Models in Medicine

To perform this task, the AI models must process free-form text through natural language processing (NLP). NLP presents challenges due to the inherent ambiguity and complexity of natural languages. Historically, NLP approaches have used simplistic models, such as the bag-of-words method, which analyzes text as a mere collection of words without considering their order or contextual relationship. While this approach suffices for basic tasks, it does not adequately capture the intricacies of human language. Consequently, researchers have worked to incorporate linguistic insights into computational models to enhance their ability to process and understand natural language.

The advent of transformer architecture, particularly with bidirectional encoder representations from transformers (BERT), has revolutionized NLP [1]. The ability of BERT to efficiently learn from extensive text corpora has significantly enhanced its contextual understanding and performance across various NLP tasks, minimizing strong inductive biases. BERT has also inspired the development of several transformer-based models tailored to specific domains, including medicine. Examples include BioBERT, ClinicalBERT, PubMedBERT, and BlueBERT [2-5]. Hence, we used JMedRoBERTa, a model specifically trained on a substantial corpus of Japanese medical research papers [6].

Large language models (LLMs), which often use architectures similar to BERT but with increased parameters and capabilities, particularly in text generation, have gained prominence. Empirical evidence from GPT-3 has demonstrated that scaling models improve performance, adhering to the scaling law in NLP [7]. The term “large” is ambiguous, as BERT can also be considered an LLM. The introduction of ChatGPT [8] and subsequent models, such as GPT-4 and PaLM (Pathways Language Model), has shown the success of LLMs across various fields, including medicine [9-11]. Despite the proprietary nature of leading models due to high training costs and safety concerns, publicly available LLMs such as LLaMA2 and OpenCalm offer opportunities for research and evaluation of their potential and limitations [12-14].

### Research Gap

The application of AI for priority estimation has been predominantly investigated in the context of emergency department (ED) triage [15,16]. Several AI models use NLP techniques to analyze medical texts [17-19], aiming to rank patients or requests to optimize the allocation of limited medical resources to those in urgent need. While these models have shown promise in improving resource allocation within ED, extending research into medical priority estimation beyond ED could further enhance patients’ quality of life, reduce hospital stay durations, and lower medical costs. Therefore, additional research is required to explore AI applications in medical priority estimation across various clinical settings.

This study provides valuable insights into both priority estimation and the broader field of medical NLP and LLM applications. Although LLMs have primarily been used for generative tasks, demonstrating innovative applications, these models underperform in scenarios requiring structured and predictable outputs. Such challenges are evident in health care settings, where integrating AI into hospital systems necessitates a high degree of precision and reliability that generative models do not consistently provide. Furthermore, current research on medical LLMs predominantly focuses on question-answering (QA) metrics [9], overshadowing the exploration of LLM potential for non-QA tasks. Emphasizing LLM applications beyond QA could reveal new practical uses in medicine.

A significant challenge in applying LLMs to our context arises from the linguistic and contextual differences between the pretraining datasets, primarily in general English, and our specific use cases involving Japanese medical terminology. This mismatch impairs the model’s understanding of specialized terms and complicates tokenization. Tokenizers, though less studied than model size and datasets, can significantly influence the performance of LLMs in non-English contexts [20]. Our study addresses this issue by evaluating and enhancing LLMs’ linguistic and contextual adaptability for diverse clinical applications.

## Methods

### Dataset

We retrospectively collected ultrasound examination requests from the waitlist system at Keio University Hospital (Figure 1) from January 2020 to March 2023. Each record comprised the requesting department, the examination slot, and the examination purpose documented by the requesting physician. In addition, records included a 6-tier priority level assigned by a board-certified radiologist during the clinical workflow, which served as the ground truth for the AI models. The criteria for determining priority levels are outlined in Textbox 1. Priority level 6 was excluded from the dataset due to its rarity (only a few records), and physicians typically communicated directly with radiologists in such cases.

Textbox 1. **Criteria for priority levels**

1) Desired before discharge if possible.

2) Required for treatment decisions.

3) Preferred early.

4) Urgently required.

5) Immediately required.

6) Emergency (excluded).

The dataset underwent 3 main preprocessing steps to ensure data quality and consistency: aggregation, cleaning, and text normalization.

#### Aggregation

Initially, records with similar request texts were aggregated using the Levenshtein distance metric, and the majority priority level was assigned to the representative record within each cluster. This aggregation was essential because the dataset contained approximately identical waitlist records for common ultrasound scenarios, such as postoperative monitoring or specific clinical pathways. Duplicates could skew sample weights during model training, and inconsistencies in priority levels could adversely affect accuracy. We aimed to reduce these risks and create a more uniform and reliable dataset for model training by aggregating similar records.

#### Cleaning

This phase involved eliminating records unsuitable for analysis. Specifically, we excluded entries with zero or invalid priority levels because these could not contribute to meaningful priority estimation. In addition, we removed records with date-specific requests (eg, “Can we schedule an ultrasound examination by May 3?”) because temporal references could bias priority estimations and present challenges for AI models during prediction. This meticulous pruning ensured the remaining dataset was relevant and suitable for accurate modeling.

#### Text Normalization

The final preprocessing step aimed to enhance textual consistency. We removed extraneous spaces and corrected punctuation errors by standardizing the text format across the dataset. This normalization was crucial for minimizing variability in model input and ensuring accurate text interpretation by AI.

After preprocessing, approximately 10% of the dataset was reserved for testing, with the remaining portion allocated for training. The dataset was divided based on referring doctors to ensure that requests from a single physician appeared exclusively in the training or test subset.

### Models

We used several pretrained models: JMedRoBERTa, Luke, OpenCalm 7B, and LLaMA2 7B [6,13,14,21], all of which are accessible via Hugging Face (Table 1) [22]. Both OpenCalm and LLaMA2 offer multiple variants with different model sizes; however, we selected the 7B model due to computational resource limitations. These 4 models were chosen based on their size and the semantic alignment between their pretraining datasets and our downstream task. Ideally, the optimal model should possess a large number of parameters and be trained on a dataset that aligns semantically and linguistically with the downstream task. However, there is often a trade-off between model size and dataset alignment. In this study, we experimented with models positioned at different points along this trade-off, providing valuable insights into how this balance can be managed for medical text classification tasks using LLMs.

**Table 1.** Model details.

Model	Number of Parameters	Language of training dataset	Category of training dataset
JMedRoBERTa	124 million	Japanese	Medical paper
Luke	562 million	Japanese	Wikipedia
OpenCalm	7 billion	Japanese	Mixed^a^
LLaMA2	7 billion	English (mainly)^b^	Mixed^a^

a Large language models are generally pretrained on diverse text data to maximize the use of their extensive parameters.

b LLaMA2 was primarily designed for English, but its training dataset included some Japanese data.

To establish a performance baseline, we also tested conventional NLP methods: support vector machine, random forest, and XGBoost (eXtreme Gradient Boosting) [23]. The same input text used for the LLMs was processed into a list of words with MeCab [24], using the mecab-ipadic-NEologd dictionary [25]. This list of words was then converted into a vector using the term frequency-inverse document frequency.

The model input adhered to the template provided in Textbox 2. We experimented with various prompts, ranging from simpler to more complex ones (such as role prompting or few-shot). Ultimately, we found that this simple prompting worked best for our task. We trained the models to predict the correct priority levels using continuous numbers (ie, regression). Training was conducted under 2 conditions: fine-tuning only the final layer and fine-tuning all layers. However, fine-tuning all layers was impractical due to the large number of parameters in OpenCalm and LLaMA2. Therefore, we used low-rank adaptation (LoRA) with parameter-efficient fine-tuning using *r*=32 [26,27]. The models were optimized using the AdamW optimizer with β₁=0.9, β₂=0.999, and a weight decay of 0.0001. The loss function used was mean squared error. Learning rates were set to 1e-5 for final-layer fine-tuning and 1e-7 for full-layer or LoRA-based tuning. Training was performed over 100 epochs, using the NVIDIA RTX A6000 GPU.

Textbox 2. **Record was fed into the models using the simple prompt.**

**[Template]**

**Input (original**): 診療科 : {診療科}; 検査項目 : {検査枠}; 依頼目的 : {依頼目的};

**Input (translated**): Department: {department}; Examination Item: {slot type}; Purpose: {purpose};

**[Example]**

**Input (original**): 診療科: 一般・消化器外科; 検査項目: 末梢血管 (両）下肢静脈; 依頼目的: 肝細胞癌術後Ｄ−ｄｉｍｅｒ上昇あり精査目的です;

**Input (translated**): Department: General and gastrointestinal surgery; Examination Item: (bilateral) veins of lower extremities; Purpose: After hepatocellular carcinoma surgery, D-dimer elevated. Needs further inspection.

**Priority level**: 2

### Evaluation

Kendall tau-b, the rank correlation coefficient, was used as the primary evaluation metric. While root-mean-squared error is a common metric for regression tasks, measuring the distance between predicted and actual values, our focus on accurately estimating the priority order for medical examinations made the alignment between predicted and actual rankings crucial. Therefore, Kendall tau-b was preferred, emphasizing the significance of ordinal relationships over quantitative discrepancies. In addition, we created the confusion matrices by rounding the continuous prediction values to the nearest integers.

In addition, we assessed the ability of the model to identify low priority (priority level=1) and high priority (priority level>=4) requests. Performance in this classification task was quantitatively assessed using the area under the receiver operating characteristic curve (ROC-AUC) and the *F*_1_-score. The thresholds were optimized individually for each classification task.

Finally, we compared the accuracy of the language models with a retrospective re-evaluation performed by a radiologist. A board-certified radiologist (MH) assigned priority levels to all records in the test dataset based solely on the same text presented to the AI models. This comparison served as a benchmark for our model’s predictions and provided valuable insights into the challenges and consistency involved in priority assignment.

### Error Analysis

We analyzed instances where the model’s predictions deviated from the actual priority levels to identify potential biases and causes of errors. The error analysis was conducted on the model that achieved the best performance, as indicated by the highest Kendall score. We extracted all samples from the test dataset with an absolute error of >1. These errors were classified as either overestimations or underestimations. Each mispredicted sample was reviewed to determine the underlying patterns or common characteristics contributing to the discrepancies. Discrepancies between the original and re-evaluation radiologist ratings were also investigated to assess the difficulty and consistency of priority estimation. In addition, we used the Shapley Additive Explanation (SHAP) [28] method to visualize the importance scores of each token in the input.

### Ethical Considerations

This study received approval from the Research Ethics Committee of Keio University Hospital (approval number: 20170018) and adhered to the Declaration of Helsinki and other pertinent ethical guidelines. All patient data were processed on the machine located inside the hospital’s secure intranet, isolated from the public internet, thereby ensuring participant privacy and confidentiality. The requirement for written informed consent was waived due to the retrospective observational nature of the study. The output of the AI models did not influence actual clinical practice.

## Results

### Dataset

**Figure 2.**
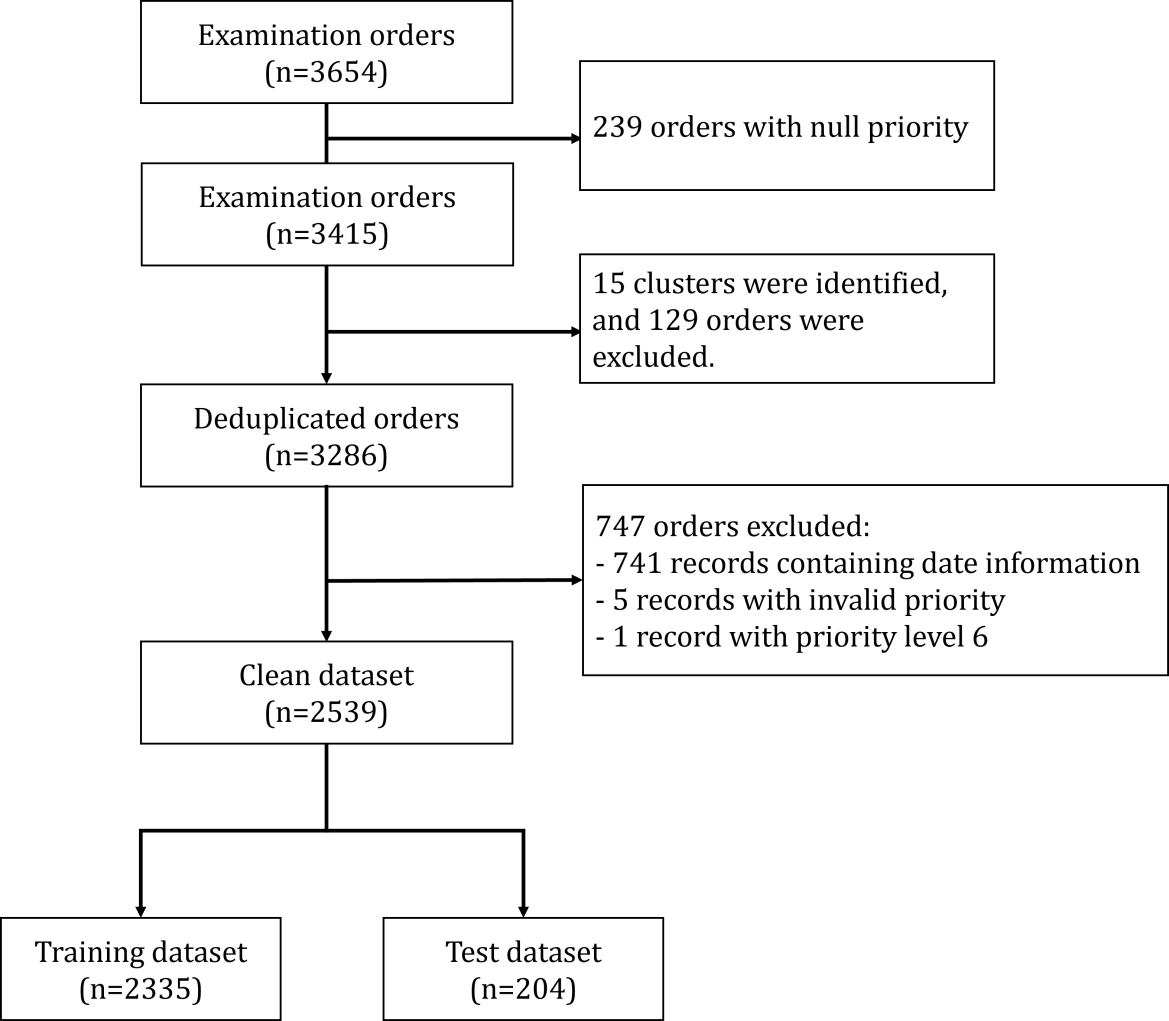
Dataset flowchart.

**Figure 3.**
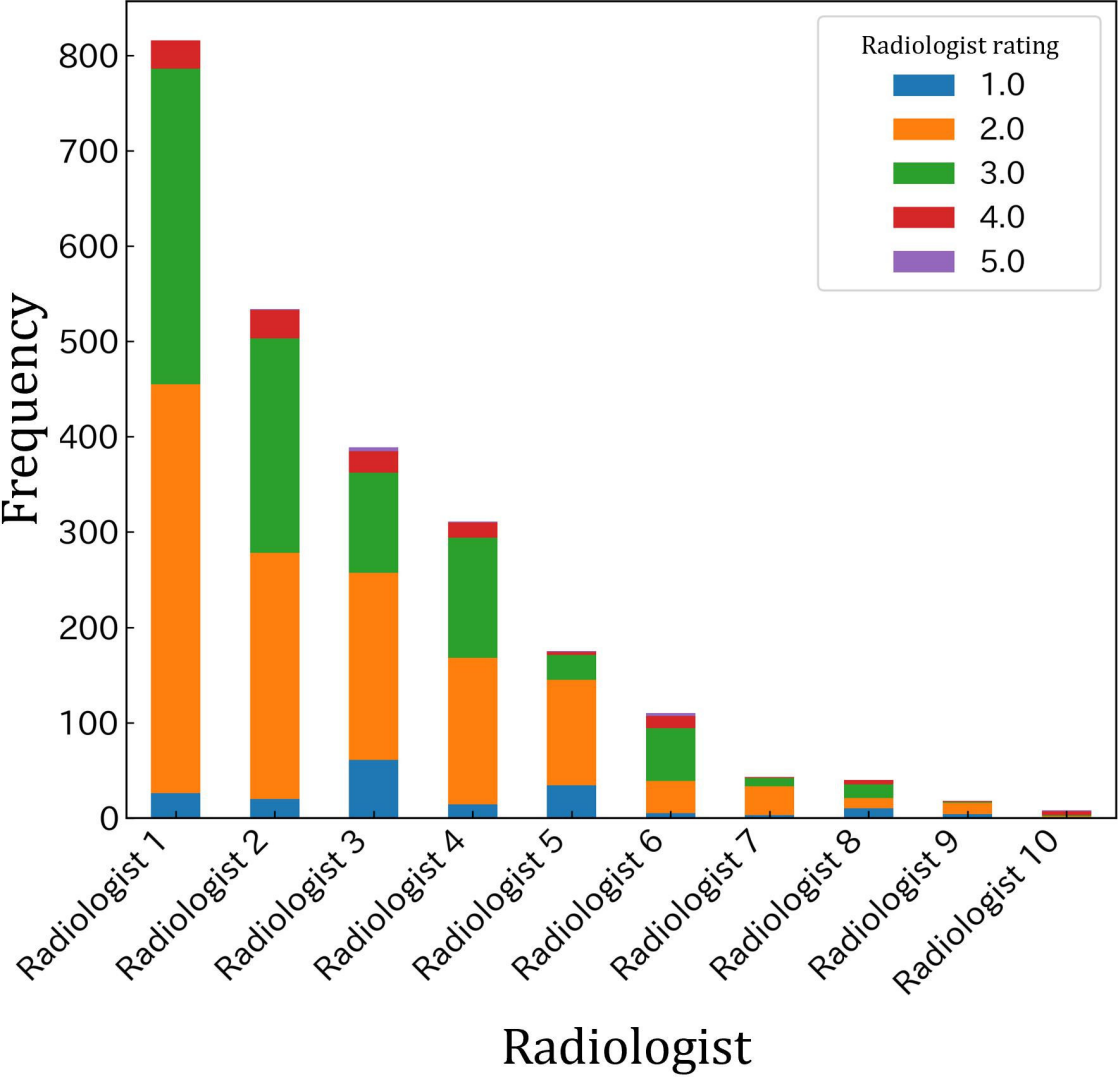
The majority of orders are assigned to priority levels 2 or 3 with little skewness between radiologists.

The initial dataset comprised 3654 ultrasound examination requests. After text similarity aggregation and data cleaning, the final dataset consisted of 2539 records (Figure 2). These were further divided into training and test datasets of 2335 and 204 records, respectively, ensuring that requests from each referring doctor appeared exclusively in either training or test subset to prevent data leakage and maintain evaluation integrity. The distribution of assigned priority levels is depicted in Figure 3. Most requests were assigned priority levels 2 or 3, while requests at priority level 5 were extremely rare. The distribution of priority levels did not exhibit significant skewness despite variability in the number of requests reviewed by each radiologist.

### Evaluation

**Figure 4.**
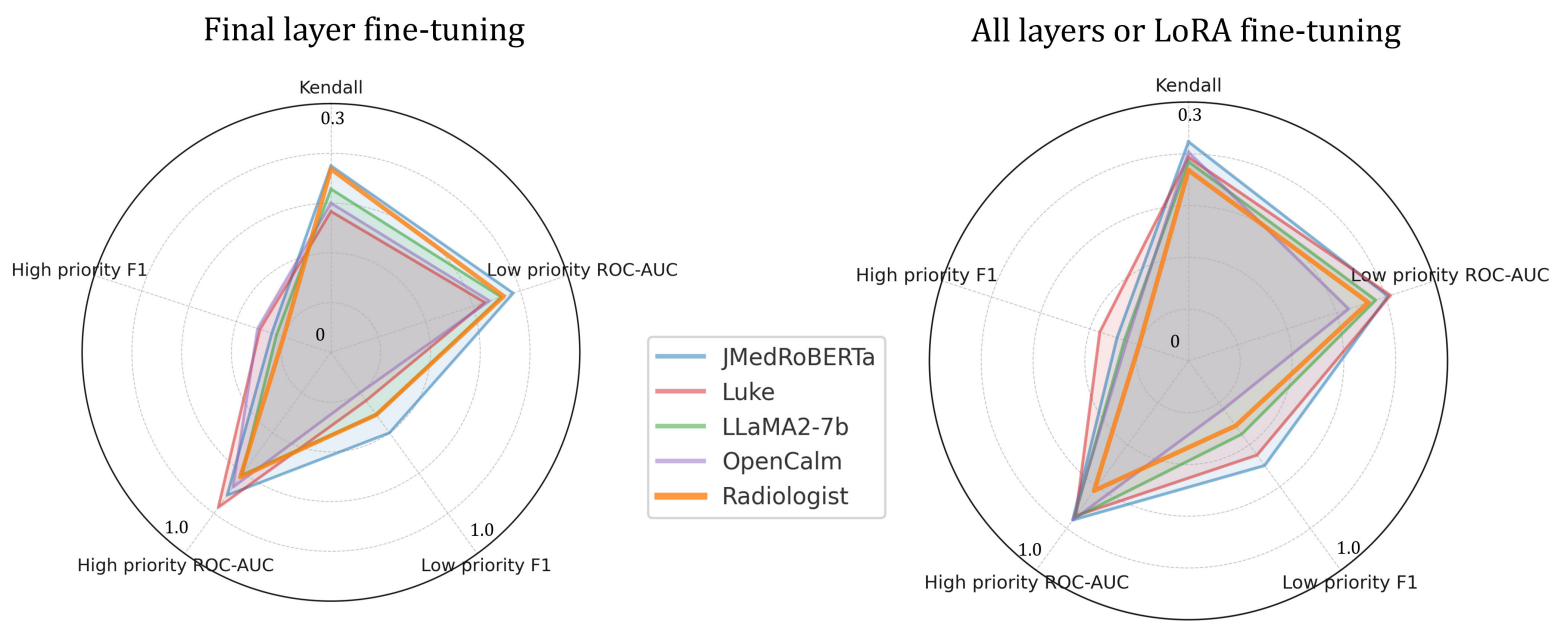
All layers or low-rank adaptation fine-tuning improves accuracy in all models, narrowing the performance gap between the medical language model and other general-purpose language models. LoRA: low-rank adaptation; ROC-AUC: area under the receiver operating characteristic curve.

Table 2 and Figure 4 show the performance of each model across different metrics. As expected, fully fine-tuned models outperformed the final-layer fine-tuned models. In particular, the fully fine-tuned JMedRoBERTa achieved the highest Kendall tau-b of 0.254. All the fully fine-tuned LLMs surpassed the baseline of conventional models, and notably, they also outperformed the radiologist re-evaluation in terms of Kendall tau-b. However, this result should be interpreted with caution, as it may reflect the inherent ambiguity of the priority estimation task, a topic that will be further discussed later. We observed that training all layers or using LoRA not only improved accuracy across all models but also narrowed the performance disparity between JMedRoBERTa and the other models. Regarding the classification tasks, JMedRoBERTa and Luke performed well, with ROC-AUC ranging from approximately 0.75 to 0.8.

**Figure 5.**
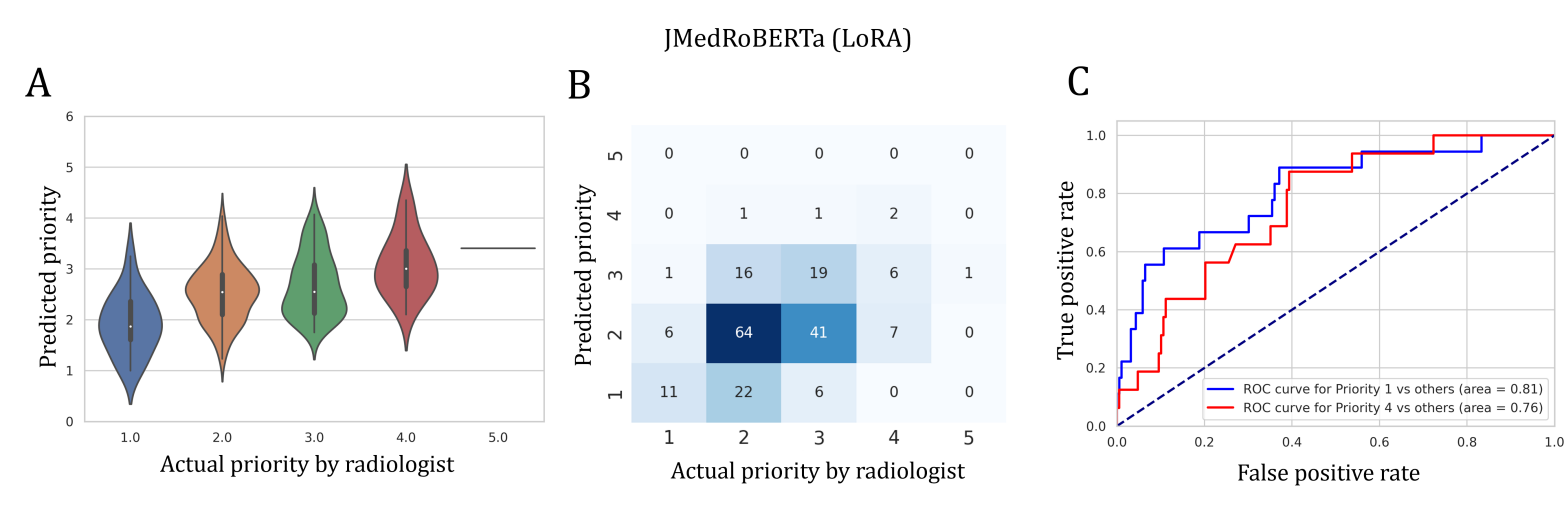
JMedRoBERTa performance. (A) The distribution of priority levels predicted by the fine-tuned JMedRoBERTa model was mostly consistent with the radiologist rating except for confusion between priority levels 2 and 3. (B) Confusion matrix also shows that the model was primarily confused by priority levels 2 and 3. (C) The model detected low (<=1) or high (>=4) priority orders at an ROC-AUC of around 0.8. LoRA: low-rank adaptation; ROC-AUC: area under the receiver operating characteristic curve.

For model-specific prediction trends, the JMedRoBERTa predictions (Figures 5A and B) revealed a trend where the distribution of AI-predicted values shifted upward as the actual priority level increased, indicating a positive correlation between AI predictions and the radiologists’ assessments.

**Table 2.** Performance metrics.

Model and fine-tuned layers	Regression	Low priority classification		High priority classification	
	Kendall	ROC-AUC^a^	*F*_1_-score^b^	ROC-AUC	*F*_1_-score^b^
JMedRoBERTa					
Final	0.225	0.77	0.40	0.71	0.25
All	0.254	0.81	0.50	0.76	0.29
Luke					
Final	0.170	0.65	0.24	0.77	0.30
All	0.236	0.82	0.45	0.74	0.36
LLaMA2-7b					
Final	0.197	0.72	0.31	0.61	0.23
LoRA^c^	0.231	0.76	0.35	0.75	0.26
OpenCalm					
Final	0.180	0.67	0.20	0.67	0.31
LoRA^c^	0.242	0.65	0.23	0.76	0.25
SVM^d^	0.167	0.61	0.27	0.49	0.08
Random forest	0.198	0.63	0.25	0.48	0.14
XGBoost^e^	0.176	0.60	0.10	0.56	0.23
Radiologist re-evaluation	0.221	0.73	0.31	0.62	0.20

a ROC-AUC: area under the receiver operating characteristic curve.

b It should be noted that these were highly imbalanced classification tasks, and therefore, *F*_1_-score tends to be lower. (A completely random classifier would yield an *F*_1_-score of around 0.1).

c Low-rank adaptation (r=32).

d SVM: support vector machine.

e XGBoost: extreme gradient boosting.

**Figure 6.**
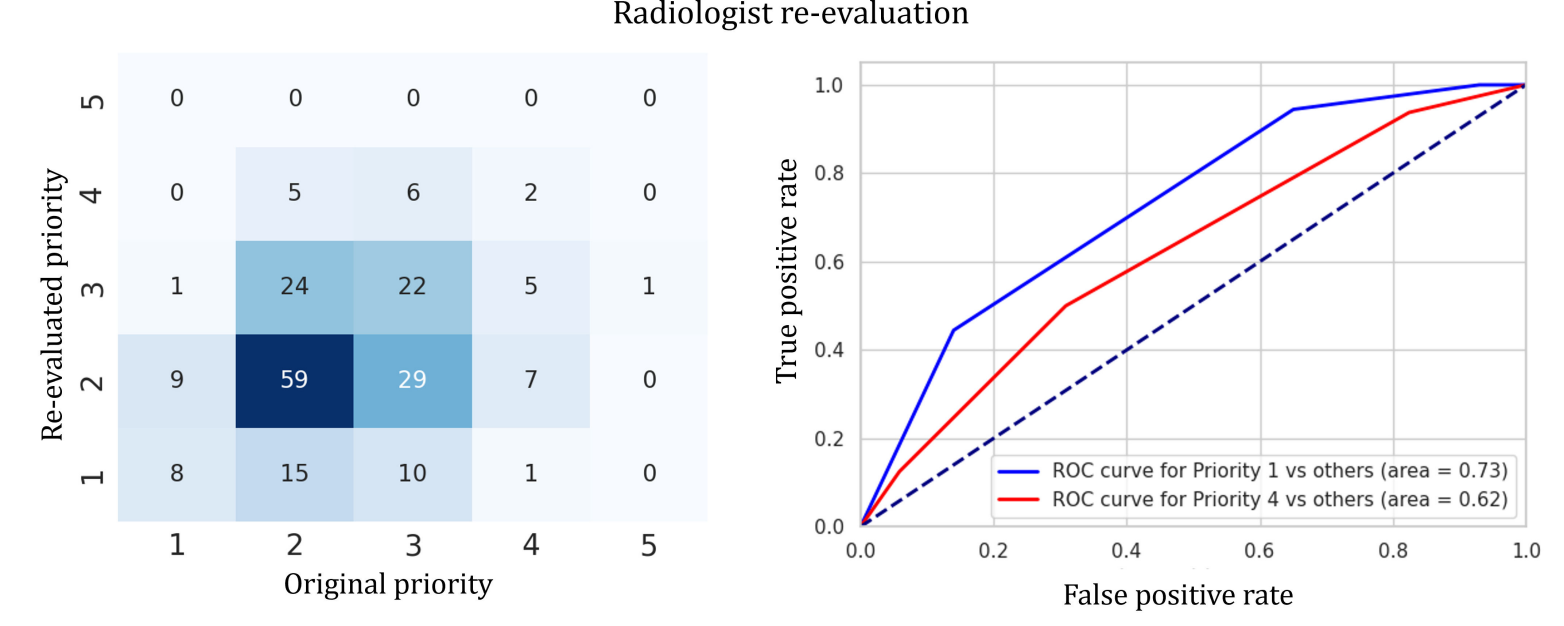
Radiologist re-evaluation performance. Even a radiologist struggled in replicating priority level 2 and 3. ROC: receiver operating characteristic.

**Figure 7.**
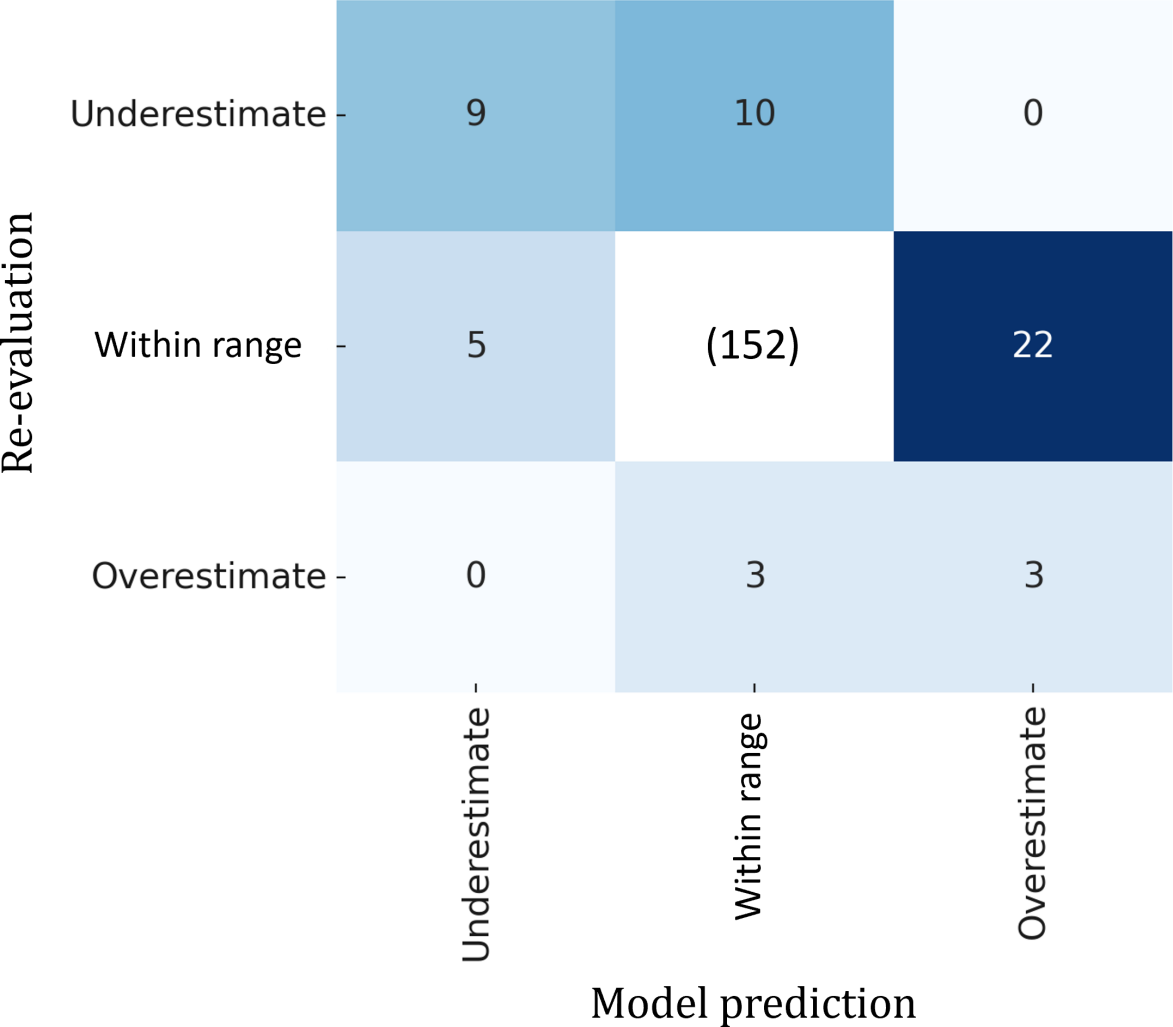
The model and radiologist tend to make similar types of errors, as seen in the upper left and lower right cells. Underestimation and overestimation are defined as a deviation of more than one level from the original radiologist rating.

A total of 39 error cases (absolute error>1.0) were identified, including 25 overestimated and 14 underestimated cases. The most common misclassification was confusion between priority levels 2 and 3, which was also observed in the radiologist re-evaluation (Figure 6). The tendency of errors made by radiologists and AI was similar (Figure 7). A more detailed analysis of these errors follows in the “Discussion” section.

## Discussion

### Principal Findings

This study used language models to predict priority levels for an ultrasound examination waitlist system. JMedRoBERTa, pretrained on a Japanese medical paper dataset, demonstrated the highest performance. Other models also performed comparably when fully fine-tuned or adjusted with LoRA. This section discusses a comparison of the performance of different models, focusing on domain and language adaptations. After that, the challenges of prioritizing tasks due to the variability of priority levels are addressed. Subsequently, the nature of the error samples is discussed, followed by the ethical and social implications. Finally, the limitations of the study and its conclusions are presented.

### Domain and Language Adaptation

Comparing the performances of the models provides insights into the influence of model size, pretraining datasets, and fine-tuning methods on the cross-domain and cross-language adaptation capabilities of LLMs. The critical factor influencing performance in this experiment was the alignment between the pretraining dataset and the downstream task. JMedRoBERTa, pretrained on a Japanese medical paper dataset, achieved superior performance despite having the smallest model size. JMedRoBERTa could focus exclusively on learning the priority assignment rules, while other models had to contend with both unfamiliar vocabulary and priority assignment rules.

However, this observation may change as the number of model parameters increases. Models pretrained on nonmedical and non-Japanese data may benefit relatively more from a larger number of training samples. In particular, as the dataset size grows, a model’s representational capacity (roughly reflected by the number of parameters) may become a more dominant factor than the similarity of its pretraining dataset, as sufficient data would enable such models to adapt to the downstream task domain.

Meanwhile, LoRA reduced the performance gap between domain-specific and general language models. Final-layer fine-tuning can be viewed as similar to zero-shot learning, as it only updates the final layer, which primarily serves to format the output from internal representations rather than contributing to text comprehension. In contrast, fine-tuning all layers, rather than just the final one, allowed the models to better adapt to the specific domain and language of the downstream task. Although full-parameter fine-tuning theoretically offers superior performance than LoRA [29], it often remains impractical due to constraints in computational resources (primarily memory capacity). Consequently, parameter-efficient tuning remains crucial for applying LLMs to medical tasks.

While our study used LoRA for fine-tuning all layers, there are other parameter-efficient tuning methods. For example, Sukeda et al [30] highlighted LoRA instruction tuning as a promising approach for adapting LLMs to Japanese medical QA tasks [30]. In addition, quantization is a popular technique that significantly reduces memory requirements while maintaining performance [31]. Our findings support the effectiveness of parameter-efficient fine-tuning, demonstrating that general-purpose LLMs can achieve capabilities comparable to those of fully fine-tuned domain-specific models.

The influence of tokenization on task performance was minimal. Only the JMedRoBRETa tokenizer could accurately recognize medical terms. Conversely, the Luke tokenizer recognized only nonmedical Japanese words, often splitting medical terms into multiple tokens. The other 2 tokenizers failed to process most Japanese characters correctly, resorting to byte fallback, where single characters were segmented into multiple tokens based on their Unicode representation. However, all models delivered comparable performances when fine-tuned. Since LoRA tuning does not change the tokenizer, it is suggested that the tokenization quality minimally affects the performance of this specific downstream task.

### Challenges in Reproducing Priority Assignments

Although the AI model outperformed the radiologist re-evaluation, this result does not necessarily indicate the superiority of LLMs in priority estimation. Instead, it highlights the inherent ambiguity of the task itself. The priority levels were originally assigned by board-certified radiologists with sufficient clinical experience; however, the relatively low interrater agreement suggests that the process is inherently subjective.

Priority assignments are influenced by various factors, some of which are only available in the real-time clinical setting, leading to discrepancies between the original and re-evaluation ratings. For instance, the number of pending orders, the availability of examination equipment, and seasonal variations such as holidays can impact decision-making. In addition, in urgent cases, physicians may directly consult radiologists or the examination department, a factor that will not be captured in the dataset used for AI training or the radiologist re-evaluation. Consequently, even experienced radiologists may find it challenging to precisely reproduce the original priority levels in a retrospective setting, and AI models face similar challenges.

To mitigate this, cases influenced by external factors should be excluded from the training dataset, with radiologists allowed to flag them. Also, enhancing request records with supplementary information would improve reproducibility. For instance, radiologists could annotate the reasons behind priority decisions, enabling AI models to learn their decision-making processes. Providing AI with comprehensive clinical notes could enrich the contextual information. While reviewing all patient charts to determine priority is impractical for humans, AI language models can process extensive text rapidly. This capability might enable AI models to exceed human performance in priority estimation.

### Error Analysis

There were 25 overestimated cases and 14 underestimated cases. The model errors can stem from 2 main sources, which are the inherent difficulty of replicating the assignment and the limitations of the model. As described previously, replicating radiologists’ priority assignments made in the clinical setting is inherently challenging, and both AI models and radiologists are affected by this uncertainty. In fact, as demonstrated in Figure 7, the model and the radiologist re-evaluation exhibited similar patterns of misclassification, with no instances where the model greatly overestimated while the radiologist greatly underestimated, or vice versa. This observation suggests that certain underlying factors (ie, inherent difficulty) may be causing both the model and the radiologist to make similar errors.

To investigate the error cases further, we examined which parts of the input text contributed most to the model’s predictions using SHAP. However, interpreting SHAP values in transformer-based models presents certain challenges. Since these models capture contextual relationships more holistically than conventional approaches, SHAP values do not always highlight clinically meaningful tokens. The same word can have highly varying SHAP values in different inputs, and common tokens appearing in all samples may absorb baseline importance, leading to misleading attributions. To partly mitigate this, we adjusted SHAP calculations to reduce the influence of shared tokens and improve interpretability. Despite these limitations of SHAP in our context, some cases yielded meaningful insights. We present the representative cases in Textbox 3, and we will discuss each case below.

Despite these limitations of SHAP in our context, some cases yielded meaningful insights. We present the representative cases in Textbox 3, and we will discuss each case below.

Textbox 3. **High-Shapley Additive Explanation tokens are shown in bold and underlined. Some cases exhibited insightful Shapley Additive Explanation values, demonstrating the model’s focus on key terms or revealing sources of misprediction.**

[Sample 1 (Original: 3, Re-evaluation: 4, AI prediction: 3.784)]

[Input (Japanese)] 診療科 : 整形外科; 検査項目 : 腹部**上腹部**; 依頼目的 : **胆嚢炎**疑い

[Translated] Department: Orthopedics; Examination Item: abdomen, **upper abdomen**; Purpose: **Cholecystitis** is suspected.

[Sample 2 (Original: 4, Re-evaluation: 3, AI prediction: 3.634)]

[Input (Japanese)] 診療科 : **産婦人科**; 検査項目 : 腹部 上腹部; 依頼目的 : **妊娠** 15 週交通事故シートベルト痕あり肝機能微増**しており, 肝損傷**疑っております FAST は陰性ですが, 右側胸部の自発痛あります御高診お願いします

[Translated] Department: **OBGYN**; Examination Item: abdomen, upper abdomen; Purpose: A 15-week **pregnant** traffic accident with a seatbelt mark. Liver enzymes are mildly elevated, **and liver injury** is suspected. FAST is negative, but she complains of spontaneous pain in the right side of her chest.

[Sample 3 (Original: 3, Re-evaluation: 2, AI prediction: 4.070)]

[Input (Japanese)] 診療科 : 心臓血管外科; 検査項目 : 頸部・甲状腺・陰**嚢**・その他表在 頚動脈ドップラー; 依頼目的 : 下行大動脈瘤**破裂**後, 仮性**瘤疑い**。**術前評価**です。

[Translated] Department: Cardiovascular surgery; Examination Item: Thyroid, Scrotum, and Other Superficial Structures / Carotid Doppler; Purpose: **Suspected pseudoaneurysm** following a **ruptured** descending aortic aneurysm. **Preoperative evaluation**.

#### Sample 1: Acute Cholecystitis Suspicion

The AI model correctly assigned a high priority to a case of suspected acute cholecystitis, focusing on the keyword “cholecystitis.” Given that ultrasound is the effective diagnostic tool for this condition and that surgical intervention may be required promptly, this prioritization aligns well with clinical expectations. This example demonstrates that when a request contains an explicit keyword suggesting a critical condition, the model can effectively capture its importance.

#### Sample 2: Traumatic Liver Injury in a Pregnant Person

For a pregnant patient involved in a motor vehicle accident with concerns of hepatic injury, the model was also assigned a high priority. SHAP analysis revealed that the model placed significant weight on the terms “pregnancy” and “liver injury,” suggesting that it successfully incorporated both the trauma and the patient’s physiological condition into its decision-making. The model’s ability to recognize such contextual factors is encouraging.

#### Sample 3: Preoperative Evaluation for Aortic Aneurysm

In this case, a carotid Doppler was requested for the preoperative evaluation of a pseudoaneurysm following a ruptured aortic aneurysm. While “aneurysm” and “rupture” typically indicate urgency, this patient appears to be stable, and the surgery is scheduled rather than urgent. If this were an urgent surgical case, it would be unlikely for the doctor to request an ultrasound examination from the radiology department. In fact, radiologists assigned a midrange priority of 2 and 3, reflecting the nonemergent nature of the request. However, the model assigned a priority of 4, overestimating the urgency. This suggests that the model may sometimes overprioritize cases based on emergency-associated keywords without fully considering the clinical context.

Overall, SHAP analysis indicates that the model performs well in straightforward cases where the primary pathology is explicitly mentioned but struggles with nuanced clinical scenarios requiring deeper contextual understanding.

### Clinical Implementation—Benefits

The current waitlist system already provides several clinical and operational advantages. Integrating AI could further enhance its efficiency by addressing the key limitations of ensuring rapid, fair, and consistent priority assignment. This section examines the benefits of the waitlist system and the role of AI in priority estimation separately.

A priority-based waitlist system offers multiple benefits. First, it improves clinical outcomes by facilitating faster ultrasound examinations for urgent cases, enabling timely clinical decisions. In addition, it can shorten hospitalization durations, especially when ultrasound examinations are critical for determining discharge eligibility. By increasing the transparency of the examination scheduling process, this system helps physicians estimate examination dates more accurately, thereby improving planning. Furthermore, effective prioritization supports better bed management and overall hospital efficiency, allowing for higher patient turnover and boosting institutional revenue.

Despite these advantages, the existing manual priority assignment process presents several challenges. Radiologists face an increased workload due to the need for subjective prioritization, leading to delays in determining priority levels. Furthermore, inconsistencies may arise from variations in clinical judgment, making prioritization less reliable.

AI offers a promising solution by automating the priority assignment process. AI models can deliver consistent, real-time estimations, improving the accuracy and objectivity of the waitlist system. By streamlining this process, AI can reduce the burden on radiologists and enhance both efficiency and standardization.

### Clinical Implementation—Challenges

However, implementing AI alone does not address all challenges. Several critical factors must be considered to ensure the successful clinical adoption of AI-assisted waitlist systems.

For AI-driven prioritization to be effectively integrated into clinical workflows, health care providers must be well-informed about its benefits and limitations to foster trust in the technology. While existing research shows a generally positive attitude toward AI in medicine [32,33], perceptions vary depending on the underlying technology, medical specialty, and cultural background [34,35]. For instance, the term “AI” encompasses a broad spectrum of technologies, from simple symptom checkers [36,37] to sophisticated LLMs [38,39]. Case studies that highlight the potential and challenges of medical AI applications will facilitate dialogue among stakeholders and accelerate the acceptance of AI in clinical practice [40].

The potential for clinically significant misclassifications remains a concern. If an urgent case is mistakenly assigned a lower priority, it could result in adverse patient outcomes. Even in situations where human evaluators might also struggle with classification, unclear responsibility could raise legal and ethical concerns regarding liability in medical decision-making.

AI models are trained on historical data, which may contain biases related to patient demographics, socioeconomic status, or institutional practices. If these biases are not addressed, they could lead to disparities in priority assignment. However, AI also offers the potential to mitigate human biases by providing consistent, data-driven prioritization. Identifying and minimizing biases through rigorous model evaluation is crucial to ensuring fairness and equity.

Integrating AI into existing hospital information systems, such as electronic medical records and order management platforms, requires substantial technical modifications. Furthermore, the costs associated with implementing, maintaining, and updating AI models may pose financial constraints for health care institutions. Assessing the cost-effectiveness and feasibility of AI adoption is critical to ensuring widespread integration.

In summary, incorporating AI into priority-based waitlist systems can enhance clinical efficiency, reduce physician workload, and improve patient care. However, addressing concerns related to user acceptance, legal and ethical responsibility, potential biases, and system integration is essential for successful implementation. Future research should focus on strategies to overcome these challenges while maximizing AI’s clinical use in resource allocation.

### Limitations

The primary limitation of this study is its focus on a single institution, which limits the external validity of the findings. Applying our model to other institutions or medical contexts would likely require retraining, as hospitals vary in specialty composition, resource allocation, and priority assessment criteria, all of which could influence model predictions. In addition, the dataset is restricted to Japanese text. Future research should aim to incorporate datasets from multiple institutions and languages. While this may present challenges due to variations in clinical practices and priority criteria, addressing these issues is crucial for evaluating the model’s robustness and generalizability. Pretraining on a sufficiently large and diverse dataset could facilitate adaptation to new institutions with minimal effort.

### Conclusions

This study demonstrates that language models can estimate examination request priorities with accuracy comparable to human radiologists and better than conventional NLP methods. Nevertheless, improvements in the reproducibility of priority rankings are required. The research also highlights the potential for adapting general-purpose models to domain-specific text through adequate fine-tuning, underscoring the flexibility and applicability of these models in specialized contexts. Further research should explore methods to address the ambiguity in priority assignment and validate the model’s performance across multiple institutions.

## Abbreviations

AI: artificial intelligence
BERT: bidirectional encoder representations from Transformers
ED: emergency department
LLM: large language model
LoRA: low-rank adaptation
NLP: natural language processing
QA: question answering
ROC-AUC: area under the receiver operating characteristic curve
SHAP: Shapley additive explanations
